# Study of thermodynamics of a thermal electron in scattering

**DOI:** 10.1016/j.heliyon.2022.e12315

**Published:** 2022-12-10

**Authors:** Saddam Husain Dhobi, Jeevan Jyoti Nakarmi, Kishori Yadav, Suresh Prasad Gupta, Bibek Koirala, Arun Kumar Shah

**Affiliations:** a Innovative Ghar Nepal, Lalitpur, 44700, Nepal; b Robotics Academy of Nepal, Lalitpur, 44700, Nepal; c Department of Physics Patan Multiple Campus, Tribhuvan University, Lalitpur, 44700, Nepal

**Keywords:** Thermodynamic properties, Thermal electron, Elastic scattering laser field, Scattering, Partition function etc.

## Abstract

This work presents the study of the thermodynamic properties of thermal electrons participating in scattering events. This is necessary because scattering with a thermal electron in presence of a laser field was not studied yet and it reduces the complexity of event measurement (differential cross-section). To study thermodynamic properties, the authors model the thermal Hamiltonian in presence of a laser field and used it to study the thermodynamic properties using the partition function. The study shows thermodynamic energy, around the target with distance at field amplitude 0.1 a.u. to 0.9 a.u. has destructive interference, above field amplitude 1 a.u. to 2.5 a.u. has superposition and at field amplitude 2.5 a.u. and 3 a.u. have coulomb potential like nature. Also, thermodynamic energy with temperature was found constant except at field amplitude 2.5 a.u. and at field amplitude 2.5 a.u. destructive interference at 10 °C and 21 °C. The thermodynamical potential at field amplitude 0.1 to 3.5 a.u. found constant and above field amplitude 3.5 a.u. increased linearly when studied with respect to temperature at 10 Å. The thermal Hamiltonian increase sharply when thermal electrons enter in 1–5 Å, slowly in 5–10 Å and beyond 10 Å constant, and the thermal Hamiltonian nature is like coulomb potential.

## 1 Introduction

Numerous applications in laboratory scattering procedures and astrophysics need for the precise determination of electron scattering cross-sections with atomic systems [1]. Finding precise wave functions for the scattered electron close to an atomic target presents a problem for the calculation of scattering cross-sections [2]. To overcomes these difficulties present work studies the wave function and thermodynamics properties of project thermal electrons and this study it may be able to reduce the difficulties of measuring scattering cross-section, authors are working to calculate scattering cross-section using the thermal wave function of electron and thermodynamic properties. To measure, the scattering cross section different matrix (S, T, R) are used among them R-matrix formalisms [3] are used to address the coupling between scattered wave functions and atomic wave functions, although due to the complexity of relativistic formalism, they are typically utilized in non-relativistic frameworks [4]. Theoretically, many researchers have studied the electron-hydrogen atom scattering in the presence of a laser field using various theoretical approaches (Rahman and Faisal, Mathur, Mittleman, Prasad and Unnikrishnan, Byron and Joachain, Dubois et al., Francken and Joachain, Bhattacharya et al., and Cionga and Florescu). Volkov state (Keldysh) can accurately depict the wavefunction of a free charged particle embedded in a laser field, but the proper description of laser-modified atomic states remains a major challenge. For instance, the typical perturbation strategy fails at conditions close to resonance as a result of a string of divergences (Jentzke et al., Francken et al.). The two-state model and the rotating-wave approximation were used by Hahn and Hertel, Gazazian, Gersten and Mittleman, Cavaliere et al., Mittleman, Pundir and Mathur, Unnikrishnan and Purohit and Mathur to depict the bound state of an atom in the presence of a resonant laser field [5]. The differential cross section of a system with a laser field and a non-thermal electron is studied by all of the authors listed, but they don't explore the Hamiltonian of thermal electron scattering in a laser field, this is the research gap and novelty of present work.

In this research, the authors looked into the thermodynamics of a thermal electron that would collide in the presence of a laser field. This is required because hot electron collision thermodynamic properties have never been explored before. There is numerous research on scattering with non-thermal electrons, according to the literature, but there is none on scattering with heat electrons. Finding the spaces around the target that change in temperature during a reaction and understanding how energy is exchanged between the target and projected particles, annihilation and creation of particles, exchange of particles, and finding the regions where scattering is best observed and measured are all made possible by studying the thermodynamical properties of thermal electrons. Due of the vast amount of unknown information in scattering, it is important to understand the thermodynamic properties of thermal projected particles.

Scattering techniques are extremely effective instruments for learning about the structural and thermodynamic features of materials, which are frequently impossible to learn using other techniques. While scattering can reveal thermodynamic features like the free energy or free enthalpy as functions of length scale, classical thermodynamics is length scale independent or applies for infinitely long length scale. Therefore, if correlations of the fluctuations as a function of time are examined, scattering techniques can provide information about the size, shape, and molecular weight of scattering particles, as well as their thermodynamic interactions with a surrounding matrix and their dynamics [6].

## 2 Materials and method

### 2.1 Quantum mechanical discrete system

Using quantum mechanics (QM) is a necessary tool for understanding how quantum in a microphysical domain, a particle behaves. QM is a logical mathematical framework that results in a thorough comprehension of several branches of physics, including plasmas, semiconductor and superconductor technology, solid-state physics, etc. Aside from that all modern physics fields, including thermodynamics, statistical physics, particle physics, optics, etc., are included in quantum mechanics (QM) even outside of the physics fields. Literature shows that thermodynamic of electron in scattering was not studies. In this work, authors first designed Hamiltonian of scattering system and using partition function different possible thermodynamic properties of thermal electron. To study the thermodynamic properties of thermal electron authors, use quantum mechanical canonical ensemble partition function, defined as the trace of the Boltzmann factor by Chester in 1954 as

(1) $$Z=tr\left(e^{-\beta \hat{H}}\right)$$

Where $\beta =\frac{1}{k_{B}T}$ and $\hat{\;H}$ is the Hamiltonian operator of thermal scattering system. This Eq. (1) is used to study the thermodynamic properties because it interconnect internal energy of system, entropy, thermodynamic potential, electronic specific heat capacity, free energy etc. of thermal electron. Jensen and Madsen, Jensen et al. designed Hamiltonian to study the differential cross section (DCS) for Laser-assisted non-thermal electron scattering in nonrelativistic spin-free case but not for thermal electron and thermodynamics properties for their work [7, 8]. This is the research gap and in present work authors design Hamiltonian for thermal electron scattering laser field to study the thermodynamics properties of thermal electron. The designed thermal Hamiltonian laser field characterized in Eq. (2) by the vector potential $\vec{A}\left(\vec{r},t\right)$ and an electric field characterized by the scalar potential φ is extended form of Eq. (10) by Maurer and Keller,

(2) $$H=\frac{{\vec{p}}^{2}}{2m}-\frac{1}{2}\left(\vec{A}\left(\vec{r},t\right).\vec{p}+\vec{p}.\vec{A}\left(\vec{r},t\right)\right)+\frac{{\vec{A}}^{2}\left(\vec{r},t\right)}{2}-\varphi +\frac{3}{2}k_{B}T$$

Here p is momentum, m is mass of electron, $k_{B}$ is Boltzmann constant, T is temperature and $\vec{A}\left(\vec{r},t\right)$ is vector potential. Eq. (2) is thermal Hamiltonian of scattering system and last term is thermal energy of electron. The vector potential of Eq. (2) is defined by Maurer and Keller as

(3) $$\vec{A}\left(\vec{r},t\right)=a\;\exp\left(i\left(\vec{k}.\vec{r}-\omega t\right)\right)=a\;\exp\left(i\vec{k}.\vec{r}\right)\exp\left(-i\omega t\right)$$

Assuming the oscillating of exponential function $\vec{k}\cdot \vec{r}-\omega t\approx -\omega t$ then from Eq. (3) vector potential depends solely on the time.

(4) $$\vec{A}\left(\vec{r},t\right)=a\;\exp\left(-i\omega t\right)$$

Here $a=\sqrt{\frac{8\pi N_{\omega }\hbar }{\omega V}}$ is vector potential amplitude, V is a volume, $N_{\omega }$ is a number of photons. For a hydrogen-like atom with charge Z, the scalar potential is $\varphi =\frac{Z}{r}$ and corresponding time-dependent Schrödinger equation (TDSE) describing [9] as

(5) $$i\hbar \frac{\partial }{\partial t}X\left(\vec{r},t\right)=HX\left(\vec{r},t\right)$$

The solution to Eq. (5) can be found in atomic physics textbooks such as the one by Bransden and Joachain. The ($X\left(\vec{r},t\right))$ is the wave function of thermal electron used to described the wave function of thermal electron in presence of laser known as modified Volkov wave function and designed as,

(6) $$X_{efT}\left(r,t\right)=\frac{1}{{\left(2\pi \right)}^{\frac{3}{2}}}\exp\left\{\frac{-i}{\hbar }\left(E_{efT}+\frac{e^{2}a^{2}}{4m}\right)t+i\frac{p_{fT}}{\hbar }.\;\left(r+\frac{ea}{m\omega }\sin\left(\omega t\right)\right)-i\frac{e^{2}a^{2}}{8m\hbar \omega }\sin\left(2\omega t\right)\right\}-k_{e}\nabla T_{efT}\;\exp\left(i\omega_{efT}t\right)$$

(7) $$X_{eiT}\left(r,t\right)=\frac{1}{{\left(2\pi \right)}^{\frac{3}{2}}}\exp\left\{\frac{-i}{\hbar }\left(E_{eiT}+\frac{e^{2}a^{2}}{4m}\right)t+i\frac{p_{eiT}}{\hbar }.\;\left(r+\frac{ea}{m\omega }\sin\left(\omega t\right)\right)-i\frac{e^{2}a^{2}}{8m\hbar \omega }\sin\left(2\omega t\right)\right\}-k_{e}\nabla T_{eiT}\;\exp\left(i\omega_{eiT}t\right)$$

Here $-k_{e}\nabla T_{e}\;\exp\left(i\omega t\right)$ thermal wave function with k_e_ is electron thermal conductivity, $\nabla T_{e}$ is change in temperature of electron [10]. Eq. (6) is thermal wave of electron before scattering and Eq. (7) is the thermal wave function of scattered electron. The designed Hamiltonian of Eq. (2) is used to study the thermodynamic energy/internal energy of thermal electron defined [1] as

(8) $$\left\langle E\right\rangle =-\frac{\partial lnZ}{\partial \beta }$$

Now on substituting the value of Z and H from Eq. (1) and Eq. (2), in Eq. (8), the thermodynamic energy is obtained as

(9) $$\left\langle E\right\rangle =-\left(\frac{3}{2\beta }-H\right)=H-\frac{3}{2\beta }$$

Eq. (9) shows thermodynamics energy of thermal electron and depend on Hamiltonian and temerpature. The variance in the energy fluctuation is obtained with the help of Eq. (1) and Eq. (2) as,

(10) $$\left\langle {\left(\Delta E\right)}^{2}\right\rangle =\left\langle \left(E-{\left\langle E\right\rangle }^{2}\right)\right\rangle =\frac{\partial^{2}lnZ}{\partial \beta^{2}}=0$$

The entropy [11] is obtained with the help of Eq. (1) and Eq. (2) as,

(11) $$S=-k_{B}\left(lnZ+\beta \left\langle E\right\rangle \right)=\frac{3}{2}k_{B}$$

Eq. (11) shows that entropy of thermal electron is constant and depend on Boltzmann constant. The Helmholtz free energy is defined as A = U − TS, where U = ⟨E⟩ is the total energy and S is the entropy is obtained with the help of Eq. (1) and Eq. (2) as,

(12) $$A=H-3k_{B}T$$

The electronic heat capacity of the thermal electron is obtained by using $C_{v}=\frac{\partial U}{\partial T}$ with the help of Eq. (8) was found constant. The thermal potential [12, 13] of thermal electron is defined as $\Omega =-\frac{1}{\beta V}\;\ln\;Z$, with the help of Eq. (1) the thermal Potential is obtained as

(13) $$\Omega =-\frac{1}{\beta V}\;\ln\;Z=-\frac{-\beta H}{\beta V}=\frac{H}{V}$$

## 3 Results and discussion

The study of thermodynamics properties of thermal electron in presence of laser field is the objectes of this work. The thermodynamics properties study in present work are thermal Hamiltonian, thermodynamic energy, thermal potential due to thermal electron, electronic heat capacity of electron and energy fluctuation. To study the nature of these with temperature and distance authors used MATLAB.

### 3.1 Thermal Hamiltonian in laser assist scattering

**Figure 1:**
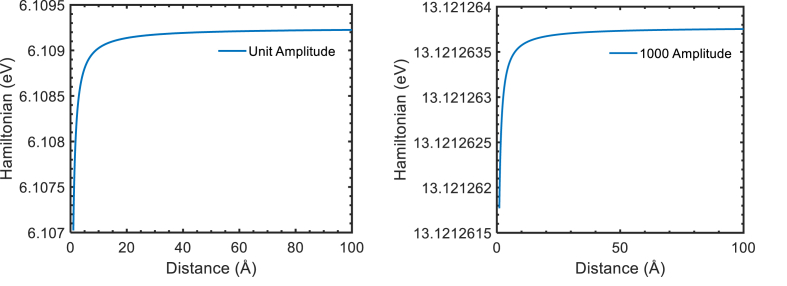
Thermal Hamiltonian at 300K with laser assisted field 1.17eV.

The designed thermal hamiltonian to study the thermodynamics properties of thermal electron in laser field with discatnce at field amplitude 1 a.u. and 1000 a.u. are shown in Figure 1 below. The nature of thermal hamiltonian was found like coulomb repuslive potential with distance at different amplitude. The computation of thermal hamiltonian Figure 1 is based on at 300K, field photon energy 1.17eV, pulse time ${\;10}^{-6}$ sec and momentum of electron 1eV.

The thermal Hamiltonian increase sharply when project thermal electron approach to target at distance 1 to 5 Å, slowly at distance 5 to 10 Å and become constant beyond 10 Å. The constant thermal Hamiltonian represent no interaction between the projected thermal electron and target. The best region to study the interaction and exchange information between projected thermal electron and target is 5–10 Å because in this region is stable and measurable. The thermal Hamiltonian below 5 Å is unmeasurable because interaction is very faster and measurement is too much complex during the collision. In addition, thermal Hamiltonian with increasing the field amplitude also. The presence in scattering with projected thermal electron assist thermal electron and increase the thermal Hamiltonian by superposing. This superposing of thermal electron and laser field cause increase in the DCS. The DCS of non-thermal projected electron to target in laser field is lower than thermal project electron to same target and same field because thermal Hamiltonian of thermal electron is greater than non-thermal. Derrickson et al. and Orzel et al., study the scattering properties of neon vapor in the ultracold temperature-range 100μK–0.5K and found collisions between neon atoms at very low temperatures (T < 1 mK) are qualitatively different than collisions at normal temperatures [14].

### 3.2 Thermodynamic energy in laser-assisted scattering

**Figure 2:**
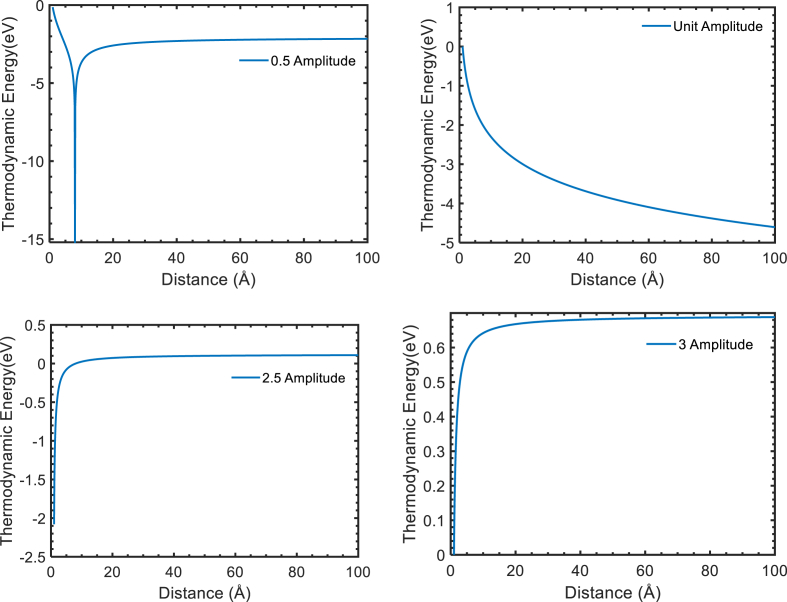
Thermodynamic energy with distance at field amplitude 0.5, 1, 2.5, and 3 a.u. respectively.

The thermodynamic energy of thermal electron in laser assisted scattering is show in Figure 2 at field amplitude 1 a.u., 0.5 a.u., 2.5 a.u., and 3 a.u. with distance. The thermodynamic energy with distance at field amplitude 0.1 a.u. to 0.9 a.u. shows down peak because of destructive interference between projected thermal electron and laser assisted. The down peak shifted left to right with distance at field 0.1 a.u. to 0.9 a.u. Except these field amplitudes, the superposing take place between projected thermal electron and laser field so no down peak is observed. In addition, the thermodynamic energy of thermal electron found greater than non-thermal electron because of superposition of wave function and $\frac{3}{2}k_{B}T$.

The thermodynamic energy at field amplitude 1 a.u. shows exponential decay with increase distance because thermal electron release the energy to the laser field. The thermodynamic energy at field amplitude 1 a.u. to 2.5 a.u. with distance has coulomb repulsive pontential like nature and shifted right to left by releasing the energy to the field. The energy release to the field by projected thermal electron decreased with distance up to field amplitude 2.5 a.u. while below field amplitude 2.5 a.u. thermal electron absorbed energy from field, as shown in Figure 2 at field amplitude 3 a.u. The thermodynamic energy of thermal electron at field amplitude 2.5 a.u. and 3 a.u. was found constant beyond 10 Å which means electron neither absorb nor emit the energy to the field in this region. Hence, concluded that the absorbance and emission of energy in the field by thermal electron depend upon the distance between target and projected particles.

In addition, the entropy for thermal scattering in presence of laser found constant while the result obtained by Maaitah et al. for Ar–Ar scattering also found constant but some they found some fluctuation in 95K to 100K [15].

### 3.3 Thermodynamical energy in laser assisted scattering with varying the temperature

**Figure 3:**
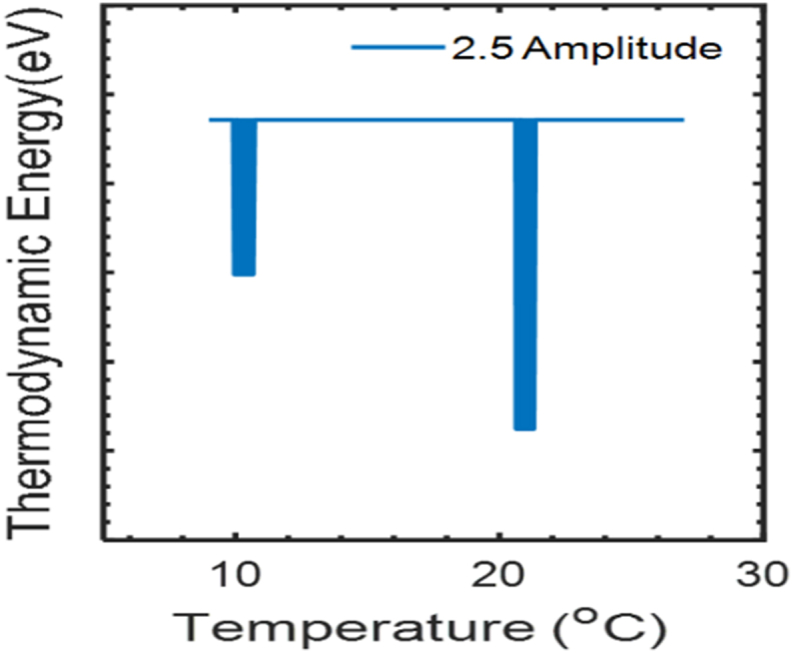
Thermodynamic energy with temperature at field amplitude 2.5 a.u.

The thermodynamic energy of thermal electron with temperature at field amplitude 2.5 a.u. is obtain in Figure 3. The observation shows thermodynamic energy at field amplitude 2.5 a.u. with temperature have destructive interference between laser field and thermal electron at 10 °C and 21 °C, and the thermodynamic energy was around 0.0247eV. In addition, the absorption of the energy from laser field by thermal electron also take place at different amplitude with temperature.

Maaitah et al study the internal energy of ^20^Ne increase with temperature in between 0.05K to 0.5K but below 0.05K authors observed fluctuation in energy and above 0.5K (normal range temperature) authors don’t study the internal energy [14].

### 3.4 Thermal potential in laser assist scattering

**Figure 4:**
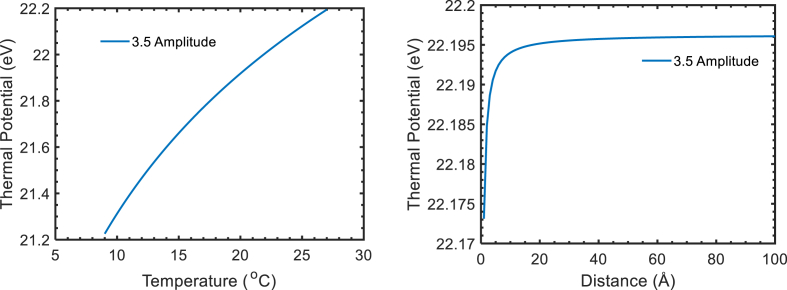
Thermal potential with temperature and distance at field amplitude 3.5 a.u.

Figure 4 represent the thermal potential in laser field with temperature and distance at field amplitude 3.5 a.u. as shown in Figure 4. The observation shows at field amplitude 0.1 a.u. to 3.5 a.u. thermal potential found constant with distance and linear with temperature at 10 Å. The thermal potential nature is like coulomb repulsive potential because of projected thermal electron and target outermost electron. The thermal potential is found greater than non-thermal potential because of superposition of thermal electron in with laser field and $\frac{3}{2}k_{B}T$. The nature of thermal potential obtained by authors has similar nature that Sahoo obtained in 2022 to study the DCS using RCCSD and RNCCSD methods [16].

To study the thermodynamical properties of thermal electron in presence of laser field we consider only coulomb potential in absence of external (perturbed) agent. This work only studies the thermodynamic properties of thermal electron like which can be used in different field of physics scattering, to supply the energy to target, to absorbed the energy from the target, etc.

## 4 Conclusion

The study of thermodynamics properties of thermal electron using quantum canonical partition function was study. The study shows that specific heat capacity and energy fluctuation of thermal electron was found constant. The thermal potential, thermal Hamiltonian and thermodynamic energy above field amplitude 2.5 a.u. was studies and study show 5 to 10 Å region is best region for measurement. The nature of thermal potential, thermal Hamiltonian and thermodynamic energy with distance was found like coulomb repulsive potential. Also, the thermal potential with temperature has linear nature at field amplitude 3.5 a.u. and thermodynamic energy with temperature has destructive interference at field amplitude 2.5 a.u. The study is beneficial to study DCS, exchange of particles and energy, variation of temperature around target, measurement of temperature of target and projected particles, annihilation and creation of particles, etc. These, study is only possible if new thermal Hamiltonian was designed. Therefore, this study is necessary to study the information hidden near the target in collision.

## Declarations

### Author contribution statement

Saddam Husain Dhobi: Conceived and designed the theoretical model; Develop theory; Analyzed and interpreted the data; Contributed reagents, materials, analysis tools or data; Wrote the paper.

Jeevan Jyoti Nakarmi; Kishori Yadav: Conceived and designed the theoretical model; Analyzed and interpreted the data.

Suresh Prasad Gupta; Bibek Koirala; Arun Kumar Shah: Contributed reagents, materials, analysis tools or data.

### Funding statement

This research did not receive any specific grant from funding agencies in the public, commercial, or not-for-profit sectors.

### Data availability statement

Data will be made available on request.

### Declaration of interest’s statement

The authors declare no conflict of interest.

### Additional information

No additional information is available for this paper.
